# Coronavirus Goes Viral: Quantifying the COVID-19 Misinformation Epidemic on Twitter

**DOI:** 10.7759/cureus.7255

**Published:** 2020-03-13

**Authors:** Ramez Kouzy, Joseph Abi Jaoude, Afif Kraitem, Molly B El Alam, Basil Karam, Elio Adib, Jabra Zarka, Cindy Traboulsi, Elie W Akl, Khalil Baddour

**Affiliations:** 1 Faculty of Medicine, American University of Beirut, Beirut, LBN; 2 Faculty of Medicine, American Univeristy of Beirut, Beirut, LBN; 3 Public Health, American University of Beirut, Beirut, LBN

**Keywords:** coronavirus, twitter, social media, epidemic, public health, pandemic, infodemic, covid-19

## Abstract

Background

Since the beginning of the coronavirus disease 2019 (COVID-19) epidemic, misinformation has been spreading uninhibited over traditional and social media at a rapid pace. We sought to analyze the magnitude of misinformation that is being spread on Twitter (Twitter, Inc., San Francisco, CA) regarding the coronavirus epidemic.

Materials and methods

We conducted a search on Twitter using 14 different trending hashtags and keywords related to the COVID-19 epidemic. We then summarized and assessed individual tweets for misinformation in comparison to verified and peer-reviewed resources. Descriptive statistics were used to compare terms and hashtags, and to identify individual tweets and account characteristics.

Results

The study included 673 tweets. Most tweets were posted by informal individuals/groups (66%), and 129 (19.2%) belonged to verified Twitter accounts. The majority of included tweets contained serious content (91.2%); 548 tweets (81.4%) included genuine information pertaining to the COVID-19 epidemic. Around 70% of the tweets tackled medical/public health information, while the others were pertaining to sociopolitical and financial factors. In total, 153 tweets (24.8%) included misinformation, and 107 (17.4%) included unverifiable information regarding the COVID-19 epidemic. The rate of misinformation was higher among informal individual/group accounts (33.8%, p: <0.001). Tweets from unverified Twitter accounts contained more misinformation (31.0% vs 12.6% for verified accounts, p: <0.001). Tweets from healthcare/public health accounts had the lowest rate of unverifiable information (12.3%, p: 0.04). The number of likes and retweets per tweet was not associated with a difference in either false or unverifiable content. The keyword “COVID-19” had the lowest rate of misinformation and unverifiable information, while the keywords “#2019_ncov” and “Corona” were associated with the highest amount of misinformation and unverifiable content respectively.

Conclusions

Medical misinformation and unverifiable content pertaining to the global COVID-19 epidemic are being propagated at an alarming rate on social media. We provide an early quantification of the magnitude of misinformation spread and highlight the importance of early interventions in order to curb this phenomenon that endangers public safety at a time when awareness and appropriate preventive actions are paramount.

## Introduction

Since December 2019, the coronavirus disease 2019 (COVID-19) epidemic has swept the world, causing significant burden and an increasing number of hospitalizations [1,2]. While public health and healthcare officials rushed to identify and contain the spread of the virus, information was spreading uninhibited over traditional and social media platforms at a strikingly rapid pace. Both the impact of the disease and the lack of information associated with it allowed medical misinformation to rapidly surface and propagate on various social media platforms. Previous reports have highlighted a similar trend during recent public health emergencies, mainly the Ebola and Zika outbreaks [3,4]. Such a phenomenon is alarming on both individual and public health levels to an extent that governing bodies are realizing its gravity and attempting to limit its effects [5-7].

Misinformation can be defined as a “claim of fact that is currently false due to lack of scientific evidence” [5]. It propagates without constraints, does not entail any curation or peer-review, and does not require any professional verifications. This makes it ideal to spread on social media and become amplified by the information silos and echo chambers of personally tailored content, particularly during times of public tension like the current COVID-19 epidemic [8]. To our knowledge, attempts to quantify misinformation during the current COVID-19 epidemic are still lacking. Hence, in this report, we seek to analyze the magnitude of misinformation that is being spread on Twitter (Twitter, Inc., San Francisco, CA) regarding the coronavirus epidemic.

## Materials and methods

Data collection

**Figure 1:**
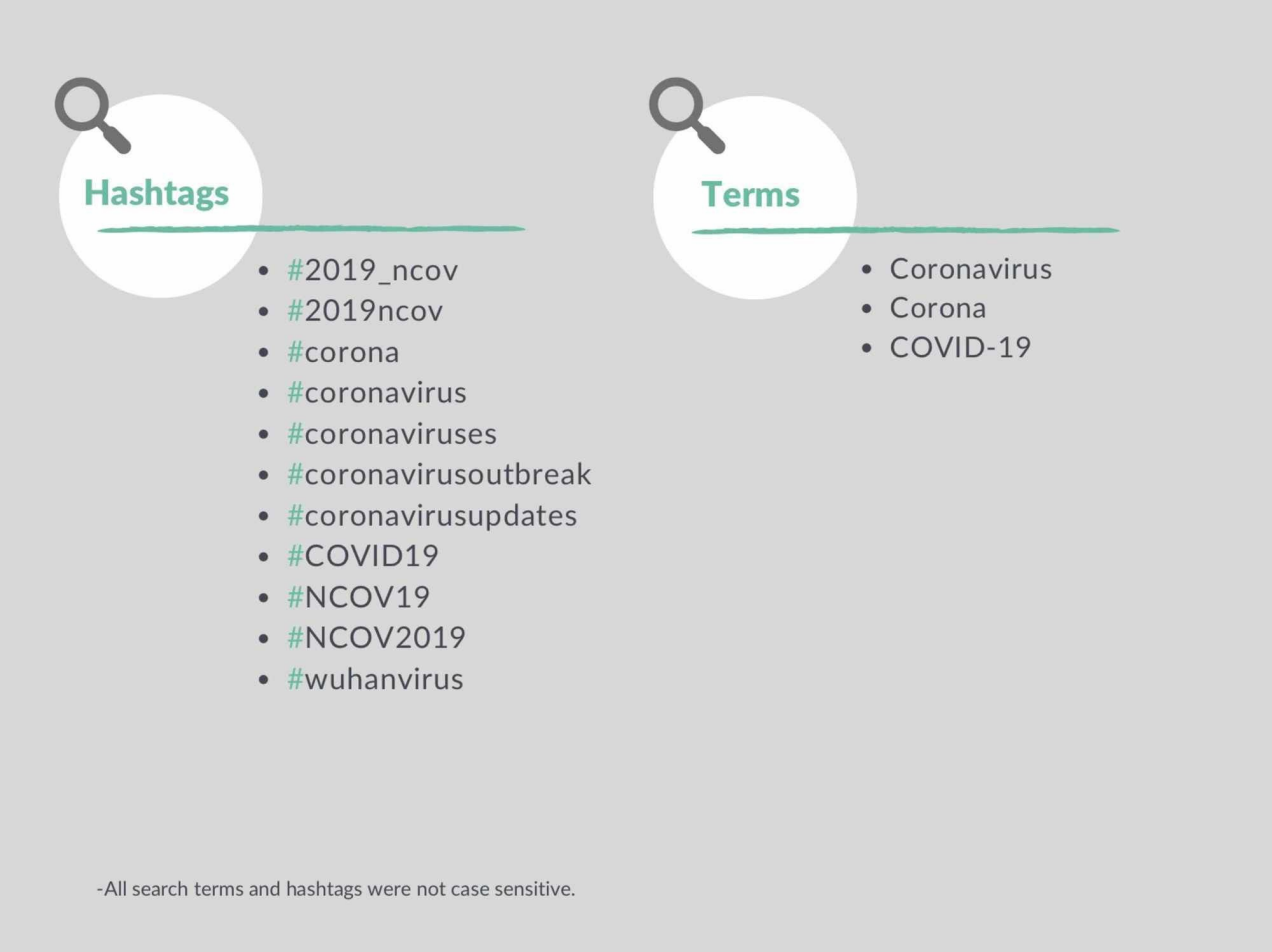
Details of the most common hashtags and search terms pertaining to the COVID-19 epidemic

We performed an online search of the Twitter social media platform on February 27, 2020. We used the Twitter Archiver add-on to search Twitter for tweets containing one or more of 11 common hashtags and three common key terms pertaining to the COVID-19 epidemic that were identified by the Symplur (Symplur LLC, Los Angeles, CA) analytical tool (Figure 1). Our search was limited to tweets in the English language and to those that initially received at least five retweets. We excluded tweets that had four or fewer retweets. We selected a random sample of 50 tweets from search terms, which yielded more than 100 tweets that fit our inclusion criteria. Samples were selected based on computer-generated random sequences.

For every individual tweet, a set of predetermined variables were collected. The different tweet/account characteristics were either automatically extracted by Twitter Archiver or manually collected from the tweets on Twitter by the authors. All data were publicly available, and hence this study did not require institutional review board approval.

User accounts were classified based on content into the following categories: informal individual/group, business/NGO/government, news outlet/journalist, and healthcare/public health/medical (Table 1). Accounts’ verified status was additionally noted. A verified account is defined as one of public interest that is deemed to be authentic by Twitter.

**Table 1:** Twitter account characteristics

Characteristics	N (%)
Informal individual/group	448 (66.6)
Business/NGO/government	37 (5.5)
News outlets/journalist	111 (16.5)
Healthcare/public health	73 (10.8)
Medical/public health	468 (69.5)
Verified Twitter account	129 (19.2)

Tweets were categorized based on content tone into the following categories: serious, humorous, and opinions. Tweets labeled as serious were those with information pertaining to COVID-19 or revolving around it, while humorous tweets consisted of jokes or memes. Tweets labeled as opinions were posts that conveyed the account's viewpoint and did not relay any novel information. Tweets were further classified based on content type into medical/public health, financial, and/or sociopolitical.

Tweets that contained genuine information regarding the COVID-19 epidemic were identified. Such information was cross-matched with the information presented by the World Health Organization (WHO), the Center for Disease Control and Prevention (CDC), peer-reviewed scientific journals, and prominent news outlets [9-12]. Tweets that included information that could be clearly refuted using one of the above-mentioned references were considered under misinformation. Tweets that could not be proven correct or incorrect by the references were designated as unverifiable information.

Statistical analysis

Descriptive statistics were conducted to analyze the Twitter accounts and tweets’ characteristics. Bar graphs were generated using Microsoft Office Excel version 16 (Microsoft Corporation, Redmond, WA). Chi-square statistic was used to calculate p-values for the association between account/tweet characteristics and the presence of misinformation or unverifiable information. Statistical significance was set a priori at a two-sided p-value of 0.05. All analyses were performed using IBM SPSS Statistics Version 26 (IBM Corporation, Armonk, NY).

## Results

Account and tweet characteristics

A total of 673 tweets were included and analyzed in this study. Most tweets were posted by informal individuals or groups (448, 66.6%), followed by news outlets or journalists (111, 16.5%). Of all accounts, 129 (19.2%) were Twitter verified accounts.

Table 2 presents the characteristics of the tweets analyzed. The majority of tweets included serious content (614, 91.2%), with information pertaining to the COVID-19 epidemic (548, 81.4%), and only 41 tweets (6.1%) included humorous content. The most frequent topic was medical/public health (468, 69.5%), followed by sociopolitical (242, 40.0%) and financial (38, 5.6%).

**Table 2:** Individual tweet characteristics

Characteristics	N (%)
Genuine content	548 (81.4)
Opinion	144 (23.0)
Tone	
Serious	614 (91.2)
Humorous/non-serious	41 (6.1)
Topic	
Medical/Public health	468 (69.5)
Financial	38 (5.6)
Sociopolitical	242 (40.0)

Misinformation and unverifiable information

**Figure 2:**
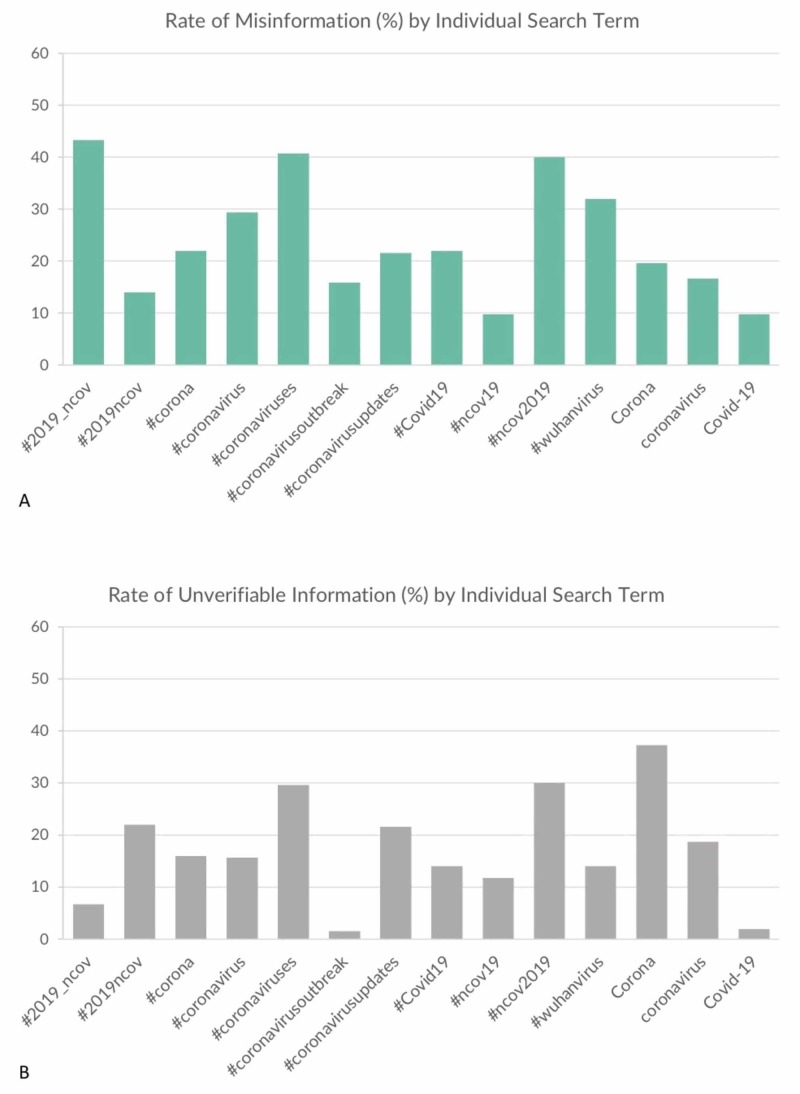
Rate of misinformation and unverifiable information by hashtags and keywords A: rate of misinformation by hashtags and keywords – “#ncov2019” had the highest rate of misinformation while “Covid-19” had the lowest; B: rate of unverifiable information by hashtags and keywords – “Corona” had the highest rate of unverifiable information while “Covid-19” and “#coronavirusoutbreak” had the lowest

In total, after excluding humorous/non-serious posts, 153 tweets (24.8%) included misinformation, and 107 (17.4%) included unverifiable information. When analyzing Twitter accounts by user category, informal personal/group accounts had more misinformation when compared to other (33.8% vs 15.0%, p: <0.001) (Table 3). Business/NGO/government, news outlets/journalists, and healthcare/public health accounts all had a lower rate of misinformation (6.1%, 18.6%, and 12.3% respectively). Furthermore, tweets posted by unverified Twitter accounts included more misinformation when compared to those posted by verified accounts (unverified account: 31.0%, verified account: 12.6%, p: <0.001). Accounts with a higher number of followers had fewer tweets with misinformation (20.1%, p: <0.001). A bigger number of likes or retweets was not associated with a higher rate of misinformation (p: 0.98 and 0.36 respectively). Lastly, the frequency of misinformation differed between hashtags/search terms, with the hashtag “#2019_nCov” having the most misinformation, and the search terms “#nCov19” “COVID-19” having the least rate of misinformation (Figure 2A).

Healthcare/public health accounts had the lowest rate of unverifiable information compared to other types of accounts (12.3%, p: 0.04). Moreover, verified twitter accounts had fewer tweets with unverifiable information (8.6%, p: 0.001). The number of followers per account, number of likes per tweet, and the number of retweets per tweet were not associated with any significant difference in terms of unverifiable information rates (p: >0.05 for all). Among search terms, the term “Corona” was associated with the highest rate of unverifiable information, while the search terms “COVID-19” and “#coronavirusoutbreak” had the lowest levels of unverifiable information (Figure 2B).

**Table 3:** Tweet and account characteristics associated with misinformation and unverifiable information ^^ ^The following numbers represent the median numbers of followers/account, likes/tweet, and retweets/tweet

Tweet/account characteristics	Misinformation, n (%)	P-value	Unverifiable information, n (%)	P-value
Informal personal/group account		< .001		0.34
Yes	123/364 (33.8)		85/349 (24.4)	
No	30/200 (15.0)		28/138 (20.3)	
Business/NGO/government		0.01		0.08
Yes	2/33 (6.1)		2/24 (8.3)	
No	151/531 (28·4)		111/464 (23.9)	
News outlets/journalists		0.03		0.25
Yes	20/107 (18.6)		21/74 (28.4)	
No	133/456 (29.2)		92/414 (22.2)	
Healthcare/public health		< .001		0.04
Yes	9/73 (12.3)		7/57 (12.3)	
No	144/491 (29.3)		106/431 (24.6)	
Verified Twitter accounts		< .001		0.001
Yes	15/119 (12.6)		7/81 (8.6)	
No	138/445 (31.0)		206/406 (26.1)	
Number of account followers		< .001		0.07
<11,045^	96/282 (34.0)		70/266 (26.3)	
>11,045^	57/283 (20.1)		43/222 (19.4)	
Number of tweet likes		0.98		0.36
<18^	80/296 (27.0)		64/258 (24.8)	
>18^	73/269 (27.1)		49/230 (21.3)	
Number of retweets		0.36		0.73
<11^	74/291 (25.4)		57/253 (22.5)	
>11^	79/274 (28.8)		56/235 (23.2)	

## Discussion

Our results raise a disturbing issue in light of the global COVID-19 epidemic plagued by a “tsunami of information” [1]. In the present study, we show that the rate of misinformation and unverifiable information is alarmingly high. Some tweets or Twitter account characteristics were seen to be associated with a higher chance of spreading unverifiable and false information. Similarly, some terms and hashtags were associated with a higher rate of misinformation compared to others. Our data quantify the pervasive spread of false or unverifiable information and provide metrics that would allow early interventions to limit its spread.

Our results are in line with those published in studies of similar recent epidemics, where social media played an important role in the propagation of misinformation [3,4,13]. However, our study has a few limitations that are worth mentioning. Firstly, our study was limited to the English language, which might have an impact on the generalizability of the findings to tweets spread by other languages. Second, the use of specific hashtags and keywords might have resulted in missing other tweets that do not necessarily utilize them. However, we selected the most common terms and hashtags that were trending on Twitter during the period of study. Third, our search timeframe was limited and hence might not capture the changing topics that evolve with the epidemic. This invites further research on the longitudinal evolution of misinformation as an epidemic or other global issue evolves and expands internationally. Nevertheless, we believe that our study offers robust and timely data on a serious challenge during the current COVID-19 epidemic and fills an important information gap.

Tweet quality (misinformation vs correct information) did not differ based on the number of likes or retweets, indicating that misinformation is as likely to spread and engage users as the truth. This implies that misinformation has the ability to spread with ease on a social media platform. This phenomenon endangers public safety at a time when awareness and appropriate preventive actions are paramount. Public health organizations, governments, and private corporations should recognize this threat and rapidly launch measures to ensure the veracity of information circulating on social media platforms. In addition to public health agencies’ endeavors to promote evidence-based information, physicians, medical associations, and scientific journals all have a role in addressing misinformation during these critical times [14]. Through global collaboration and multidisciplinary partnerships, misinformation could be contained, debunked, and replaced by facts presented by medical publications and accurate information pertaining to the topic.

## Conclusions

Medical misinformation and unverifiable content pertaining to the global COVID-19 epidemic are being propagated at an alarming rate on social media. We provided an early quantification of the magnitude of misinformation spread and highlighted some of the characteristics that might be associated with it. Interventions from multiple stakeholders are essential in order to curb this phenomenon and harness the power of social media to disseminate reliable and vetted information.
